# The effectiveness of a “EspaiJove.net”- a school-based intervention programme in increasing mental health knowledge, help seeking and reducing stigma attitudes in the adolescent population: a cluster randomised controlled trial

**DOI:** 10.1186/s12889-022-14558-y

**Published:** 2022-12-24

**Authors:** Rocío Casañas, Pere Castellvi, Juan-José Gil, María Torres-Torres, Jesica Barón, Mercè Teixidó, Hernán María Sampietro, Marta Díez, Raúl Fernández, Raquel Sorli, Patricia Siñol, Francisca Jurado, Regina Carreras-Salvador, Davinia Vazquez, Sandra Gonzalez, Maria Isabel Fernandez-San Martín, Antonia Raya-Tena, Rosa Alvarez, Isaac Amado-Rodriguez, Luis Miguel Martín- López, Jordi Alonso, Lluís Lalucat-Jo

**Affiliations:** 1 Research Department, Associació Centre Higiene Mental Les Corts, C/ Numància, 103-105, Bajos, 08029 Barcelona, Spain; 2 Department of Medicine, Universitat Internacional de Catalunya, Campus Sant Cugat. C/ Josep Trueta s/n, Sant Cugat del Vallés, 08195 Barcelona, Spain; 3 Child and Juvenile Mental Health Centre of Les Corts- Sarrià Sant Gervasi, Associació Centre Higiene Mental Les Corts, C/Montnegre 21, 3a Planta, 08029 Barcelona, Spain; 4 Mental Health Area of Les Corts- Sarrià Sant Gervasi, Associació Centre Higiene Mental Les Corts, C/Via Augusta 364-372, 4a Planta, 08017 Barcelona, Spain; 5 Activament Catalunya Associació, C/ Rocafort, 242 Bis, 3R B, 08029 Barcelona, Spain; 6 Child and Juvenile Mental Health Centre of Ciutat Vella- Sant Martí, Instituto de Neuropsiquiatria y Adicciones del Parc de Salut del Mar (INAD), Consorci Parc de Salut Mar, C/ Ramon Turró, 337, 339, 08019 Barcelona, Spain; 7 Gerència d’Atenció Primària Barcelona Ciutat, Institut Català de la Salut (ICS), C/Balmes, 22, 08007 Barcelona, Spain; 8 Instituto de Neuropsiquiatria y Adicciones del Parc de Salut del Mar (INAD), Consorci Parc de Salut Mar, Passeig Marítim, 25-29, 08003 Barcelona, Spain; 9 Institut Hospital del Mar d’Investigacions Mèdiques (IMIM), CIBERESP, C/ Dr. Aiguader, 88, 08003 Barcelona, Spain

**Keywords:** Mental health literacy, Adolescence, School, Intervention, Mental health, Promotion, Prevention, Stigma

## Abstract

**Background:**

The aim of this study is to evaluate the short- and long-term effects of the universal mental health literacy intervention “EspaiJove.net” in increasing mental health knowledge, help seeking and reducing stigma attitudes in the adolescent population. We also examine whether these effects depend on the intervention intensity.

**Methods:**

A clustered school-based randomised controlled trial (cRCT) design. Subjects: 1,298 secondary pupils aged 13 and 14 were recruited from 18 schools in Barcelona (Spain) between September 2016 and January 2018. Intervention: Three programmes were assessed: 1) Sensitivity Programme (SP; 1 h); 2) Mental Health Literacy (MHL; 6 h); 3) MHL plus a first-person Stigma Reduction Programme (MHL + SR; 7 h); 4) Control group (CG): waiting list. Outcome measures: 1) MHL: EspaiJove.net EMHL Test (First part and Second Part); 2) Stigma: RIBS and CAMI; 3) Help-seeking and use of treatment: GHSQ. Analysis: The data was collected at baseline, post-intervention and 6 and 12 months later. An intention-to-treat analysis and imputation method was used to analyse the missing data. Intervention effects were analysed using multilevel modelling.

**Results:**

One thousand thirty-two students were included (SP = 225; MHL = 261; MHL + SR = 295 and CG = 251). The MHL and MHL + SR interventions showed short- and long-term an increase in knowledge compared to SP and CG, but no significant change post-intervention or over time (First part *p* = 0.52 and Second part *p* = 0.62) between intervention groups and CG. No significant changes were found in stigma scores post-intervention or over time (CAMI *p* = 0.61 and RIBS *p* = 0.98) or in help-seeking scores (parent *p* = 0.69; teacher *p* = 0.23 and healthcare professional *p* = 0.75). The MHL + SR intervention was the best valued and recommended (*p* < 0.005).

**Conclusions:**

The three interventions of the EspaiJove.net programme (SP, MHL and MHL + SR) seem not to be effective in terms MHL, Stigma and help-seeking behaviours. The contact with a person who has experimented mental illness first-hand did not reduce stigma attitudes. Further research should deal with the heterogeneity of MHL interventions (concept, duration and measures) and identify which components of stigma interventions are effective.

**Trial registration:**

ClinicalTrials.gov identifier: NCT03215654 (registration date 12 July 2017).

**Supplementary Information:**

The online version contains supplementary material available at 10.1186/s12889-022-14558-y.

## Background

Adolescence is a period of multiple physical, emotional and social changes and is a stage of high vulnerability for developing mental health problems [1]. Epidemiological data show that 75% of all people suffering from a mental disorder have experienced the onset by the age of 25 [2, 3] and 50% during adolescence [4]. According to the World Health Organisation [1], half of mental disorders begin before the age of 14, but most cases are not detected or treated and therefore tend to extend into adulthood [5].

Studies show that stigma and lack of mental health literacy are associated with mental disorders and delays in seeking help [6–9], with only a minority of young people experiencing a diagnosable mental disorder accessing professional help.

The promotion of mental health and the prevention of mental disorders and their consequences is one of the main goals in public health [10]. Therefore, international institutions [11, 12] recommend the implementation of comprehensive, integrated and evidence-based programmes for early detection and improvement of the mental health of children and young people, involving sectors other than health, such as education [13]. They also recognise the value of educational centres as an ideal environment to act for the benefit of health and emotional well-being [14–16]. At this moment, countries such as Canada [13], Australia [17] United Kingdom [18] and Japan [19] have initiated educational courses related to MHL nationwide.

Mental Health Literacy (MHL) was first defined [20] as ‘knowledge and beliefs about mental disorders which aid their recognition, management or prevention’ which includes aspects such as: 1) the ability to recognise specific disorders; 2) knowledge and beliefs about risk factors and causes; 3) knowledge and beliefs about self-treatments; 4) knowledge and beliefs about professional help available; 5) attitudes which promote recognition and appropriate help-seeking; and 6) knowledge of how to seek mental health information [8]. Other more current definitions include aspects such as: 1) understanding how to obtain and maintain positive mental health; 2) understanding mental disorders and their treatments; 3) decreasing stigma related to mental disorders; and 4) enhancing help-seeking efficacy [21].

A number of MHL interventions for adolescents in a school context have been developed in recent years in several countries [22–25]. These interventions suggest an improvement in mental health knowledge, an increase in the self-recognition of mental disorders and facilitating monitoring and help-seeking and, to a smaller degree, stigmatizing attitudes and social distance. On the other hand, interventions which used first-person experience showed an increase in mental health knowledge, but these are not more effective in reducing stigma than education interventions, and the results suggest to identify which intervention components are more effective [18, 25, 26]. However, they suggest that further research with objective measurement tools and using more rigorous methodological designs (randomised controlled trials—RCTs) is needed to confirm these findings [22, 23].

The EspaiJove.net: a space for mental health (EspaiJove.net) programme is a universal MHL programme which aims to promote mental health, prevent mental disorders, facilitate help-seeking behaviours and eradicate related stigma among secondary school students (aged 11 to 18) within the Spanish context. The programme integrates a multi-modal intervention that combines taught classes and training activities among schools with the use of Information and Communication Technology (ICT) such as the website www.espaijove.net and online consultation [27, 28]. The EspaiJove.net programme includes three intervention modalities that differ in the duration (1 h, 6 h and 7 h) and content, according to the needs and availability of the schools. A first intervention designed to provide a first contact with mental health topics (sensitive program; 1 h) with the objective of providing a space where you can talk about mental health with youth. A second intervention of greater intensity and focused on mental health literacy (6 h) and a third intervention which includes working on the stigma in collaboration with expert entities in the field (7 h). The program is supported by the Department of Health of the Generalitat of Catalonia, and it is based on the recommendations of the World Health Organization (WHO) and European Commission [12] to implement programs to improve the mental health of young people in educational centers. These three interventions are being carried out in students from 13 to 18 years of age from educational centers of Barcelona since the academic course 2013.

To the best of our knowledge, this is the first study to evaluate the short- and long-term effectiveness of a universal MHL programme in Spain. Furthermore, we have evaluated three MHL programmes of different duration.

The main objective of the study was to evaluate whether the three interventions of the “EspaiJove.net” programme have a short- (post-intervention) and long-term (6 and 12-month follow-up) impact on increasing MHL among students. A secondary objective was to analyse the impact of these three interventions of different intensity on increasing MHL, help-seeking behaviours and reducing related stigma.

The main hypothesis of the study was that the participants in the intervention groups (Sensitivity Programme (SP), MHL programme and MHL + SR programme) would increase their knowledge on mental health and mental disorders, increase help-seeking behaviours and reduce the stigma associated with mental illness compared to a waiting list control group immediately after 2 weeks, 6 months and 12 months post-intervention.

## Methods

### Design and setting

A multicentre, school-based clustered randomised controlled trial (cRCT) was undertaken in 18 schools in Barcelona, Spain. The full study protocol is described in a previous article [28]. The study was designed and reported in accordance with the standards of CONSORT 2010 extension to cluster randomised controlled trials [29]. The trial has also been registered on the Clinical.Trial.gov register (NCT03215654; registration data 12/07/2017).

### Participants

Participants were 13 and 14 year-old students at public or private secondary schools (attending the 3^rd^ year of E.S.O (Compulsory secondary education) or 9^th^ grade) in the city of Barcelona, Spain, who consented to participate in the study. Exclusion criteria at the school level included: (1) special education schools; (2) schools whose official language is not Catalan/Spanish; and (3) schools that did not consent to participate in the study. Exclusion criteria at the participants’ level included: Students who (4) had attended an MHL programme prior to the study; (5) had special educational needs attending any school; and (6) did not understand Spanish and Catalan.

### Sample size

The same size calculations were used based on a previous RCT of MHL [30], assuming that we wanted to be able to identify a minimal difference of 0.18 effect size in MHL scores after the intervention. To detect differences in each group with one-way analysis of variance (ANOVA) that corresponds to a small effect size (0.18), with an alpha risk of 0.05, and a power of 80%, the adequate sample size required for this study in each group was 85 students (*n* = 340). Assuming an attrition rate of 20% during the 12 months of follow-up after the intervention, we estimated that a total sample of 408 students was required. The study of Naylor et al. [30] assumed no intraclass correlation. Given the particularity and complexity of our study, which analyzes 4 groups, 9 outcomes in a RCT by clusters (school-*based), and given the lack of similar references, a design effect of 3.45 (340 * 4), which would imply in a study with 18 participating schools of 50 students, assuming an ICC of 0.05.

### Recruitment process and randomisation

The recruitment process for the trial began in September 2016. All eligible secondary schools within Barcelona city were contacted and encouraged to participate in the study. Emails providing information about the study were sent to all schools, 18 of which, representing 9.4% of schools in Barcelona, agreed to participate and to permit accessibility to the schools and students, while being committed to the continuity of the project. Once a school agreed to participate, consent letters were sent to the director of the school, and the school was in charge of sending it to the parents/legal guardians of all participating students.

Out of all the schools that agreed to participate in the study, a cluster randomisation by school was conducted into three experimental groups and one control waiting list, stratifying according to the number of classes in the school (≤ 5 classes and > 5 classes). The randomisation by cluster (schools) was carried out through a computer program, and the group assignment was 1:1:1:1. The randomisation process was carried out by external research professional. Interviewers were blinded at each assessment.

An Informed Consent Form (for adolescents and parents) was required for all participants prior to their involvement in the study. The confidentiality of the participants (adolescents) was protected using an encryption key for any personal details within the data (school 1–18, class 1–6, identification number 1–34). The key was stored separately. One teacher was the unique person responsible for keeping the identification number and corresponding student. Ethical approval was granted by the Fundació Unió Catalana Hospitals (CEIC 15/33).

### Procedure

Pre-treatment questionnaires were completed approximately two weeks before the intervention by all students (intervention and control groups). Two researchers were responsible for supplying each student with a questionnaire for completion during each assessment within the study. Collected data was imported using a teleform format program onto a computer database. All participants completed the questionnaires at school during a tutorial class and it lasted one hour.

A second evaluation was performed approximately two weeks after the intervention or one month after the baseline assessment in the control group. A third and fourth evaluation were examined at 6 and 12 months respectively.

### Intervention

The intervention was described in the manuscript protocol [28]. Table 1 describes the contents of the Espaijove.net among the different intervention groups compared in this study: 1) Sensitivity programme (SP; 1 h); 2) Mental health literacy programme *(*MHL; 6 h) and 3) MHL plus Stigma Reduction (MHL + SR; 7 h). The programmes cover the following topics:

- Sensitive program (SP): this program aims to increase knowledge about the definitions of mental health and mental disorders (emotional management). It is a first contact with mental health topics (1 h; session 1).
- Mental Health Literacy (MHL): this program aims to promote mental health wellbeing, facilitate help-seeking behaviours on their own, raise awareness of the consequences of risky behaviours (substance abuse), identify mental health disorders (i.e., anxiety, depression, eating and behaviours disorders, psychotic disorder, self-harm and suicidal behaviours) and when/where to seek treatment, so as to eventually prevent and detect mental health-related problems early (6 h; session from 1 to 6).
- Mental Health Literacy plus Stigma Reduction (MHL + SR): Additionally, a person who has experienced mental illness first-hand speak about his/her personal life experience with the students so as to aim to reduce any related stigma (7 h; session from 1 to 7).

**Table 1:** Contents of the EspaiJove.net programme in the different intervention groups

Session	Workshop Contents	SP (1 h)	MHL (6 h)	MHL + SR (7 h)	Comparison group
1	Concepts of mental health (MH) and mental disorders (MD). Emotional management	x	x	x	Waiting List
2	Healthy and risk behaviours in MH		x	x	
3	Social skills and antisocial behaviours, bullying and cyberbullying		x	x	
4	Anxiety, depression and self-harm and suicidal behaviours		x	x	
5	Eating and behavioural disorders		x	x	
6	Substance abuse. Psychotic disorders		x	x	
7	Stigma Self-experience in MD and MH presentation of an activist of a voluntary member of Activament Catalunya Associació (http://www.activament.org/es)			x	

*Abbreviations: MHL* Mental Health Literacy Programme, *MHL* + *SR* Mental Health Literacy Programme plus Stigma Reduction, *SP* Sensitivity Programme

Students from the control group were waiting list, and they received the MHL + SR programme after the 12-month follow up, thus during the next academic year of 2018–2019.

The intervention was delivered by five mental health nurses from four Child and Adolescent Mental Health Services (CAMHS), with vast experience in the treatment of children and adolescents. Nurses received previous training in relation to contents of the six modules of EspaiJove.net programme. The training period was 24 h (12 h of theory and 12 h of practice).

Each intervention (SP, MHL and MHL + SR) was delivered to students during a tutorial class (one hour/week). The format of the intervention was an instruction of the content of the six modules of the EspaiJove.net programme supported by prezi format presentations. The nurses encouraged the participation of the students through questions.

### Outcomes

Socio-demographic variables such as age, gender, nationality, city/town; district of residence or postcode, were considered potentially confounding variables.

#### Mental health knowledge

**EspaiJove Mental Health Literacy Test (EMHL Test)** [31] is a self-reported questionnaire based on the thematic contents of the EspaiJove.net school programme. The EMHLT consist of 35 items, using two response formats: (i) the first part consists of a binary choice format (yes/no) for the recognition of mental disorders from a list of 15 different diseases; (ii) the second part contains 20 multiple choice questions with four possible answer options, in which only one is correct. The score for each part of the test ranges from 0 to 10. Higher scores in the first part mean more recognition of mental disorders. Higher scores in the second part indicate more knowledge about mental health. The first part of EMHL test has a test–retest reliability of 0.578, and a Cronbach’s α of 0.744. The second part of EMHL test has a test–retest reliability of 0.422, and a Cronbach’s α of 0.615. The EMHL test is a relevant measure for assessing MHL in adolescents into Spanish context with acceptable validity and reliability.

#### Stigma. Behaviours and attitudes towards mental health

**Reported and Intended Behaviour Scale (RIBS)** consist of eight items. The first four items are designed to assess prevalence (past and current) of behaviour in each of the four contexts (1. living with; 2. working with; 3. living nearby; and 4. being in a relationship with someone with a mental health problem) while items 5–8 ask about intended (future) behaviour within the same contexts [32]. For the purposes of this study, we selected four items from 5 to 8. The total score is obtained from a sum of the total answers ranging from 4 to 20; the higher the score, the higher the stigma associated to mental health problems. This scale has high test–retest reliability (0.75) and acceptable to good internal consistency (Cronbach’s alpha = 0.72 -0.81) [32].

**The Scaling Community Attitudes toward the Mentally Ill (CAMI)** [33] is an instrument for the systematic description of the attitudes of the community towards mentally ill people, which consists of 40 items divided into four dimensions (Authoritarianism; Benevolence; Community mental health ideology and Social restrictiveness). Only the “Authoritarianism” 10-item dimension in the Spanish version is used [34]. All items are scored on an ordinal scale (5–1), respectively, ranging from 10 to 50 for each factor; the higher the score, the higher the stigma associated to mental health problems. The internal consistency was α 0.86 for the first evaluation and 0.909 for second evaluation. The values of the intraclass correlation coefficient ranged from 0.775 to 0.339 in the item by item analysis, and between 0.88 and 0.81 in the subscales [34].

#### Help-seeking and use of treatment

The instrument selected consists of two parts: (i) the first part is an ad hoc questionnaire where we ask whether the person has received psychological and/or medicinal treatment for an MH problem at any time in her/his life; and (ii) the second part includes the first item from the Spanish version of the **General Help-Seeking Questionnaire (GHSQ)** for measuring help-seeking behaviour from different sources when a student is experiencing a mental health problem [35, 36]. The sources of help evaluated were: friend, parent, teacher, mental health professional and ‘no one’. They were evaluated at baseline and at 6 and 12-month follow-up. Higher scores indicate greater likelihood of intending to find help for a problem (range 1–7).The questionnaire has a good internal consistency (Crombach ‘s alpha = 0.70) [35].

#### Acceptability and satisfaction

We developed an ad hoc short questionnaire (4 items, 3-point Likert scale) for assessing acceptability and satisfaction to measure the degree of receptivity of the adolescents to the interventions proposed (interesting, useful, resolved doubts and recommended).

### Statistical analysis

A description of the sociodemographic variables of the sample at baseline was done through a univariate analysis of the selected variables from the total sample and stratified by type of intervention. Then, to evaluate the effectiveness of all programmes for each outcome after the intervention, an unadjusted bivariate primary analysis was performed. Data scores after the intervention were analysed with linear mixed models, taking into account visit, school and group. This statistical model is appropriate for longitudinal clustering analyses since this study design (schools) allowed us to consider the individual data correlation within the same cluster group [37]. A sensitivity analysis was done adjusted for: gender, nationality, psychological help or medications for mental health. The intraclass correlation coefficient (ICC) was also shown for each model.

To measure the effect size between groups comparisons we estimated Cohen’s d with R (effectsize) package [38]. It provides functions for estimating the common indices of standardized differences such as Cohen’s d (cohens_dfunction) [39].

Analyses were based on an intention-to-treat analysis and multiple imputation analysis (MIA) was used to take account the missing data. Missing data were accounted for through Predictive Mean Matching with ten imputations, each of which has five interactions. The evaluation of the parameters of each imputation was carried out according to Rubin’s rules [40]. Finally, a comparative analysis at baseline between subjects with complete information versus subjects with dropouts (those who had an imputed value in any of the evaluations: post-intervention, 6 and 12 months follow up) was performed. Also, sensitivity analysis comparing complete cases analysis versus MIA analysis was performed (data not shown).

The statistical analyses were performed using R3.6.1 software (https://www.r-project.org/).

## Results

Recruitment of participants took place between September 2016 and January 2018. Eighteen schools in the city of Barcelona (Spain) participated in the study, 16 (88.9%) of which were public and two (11.1%) private.

**Fig. 1:**
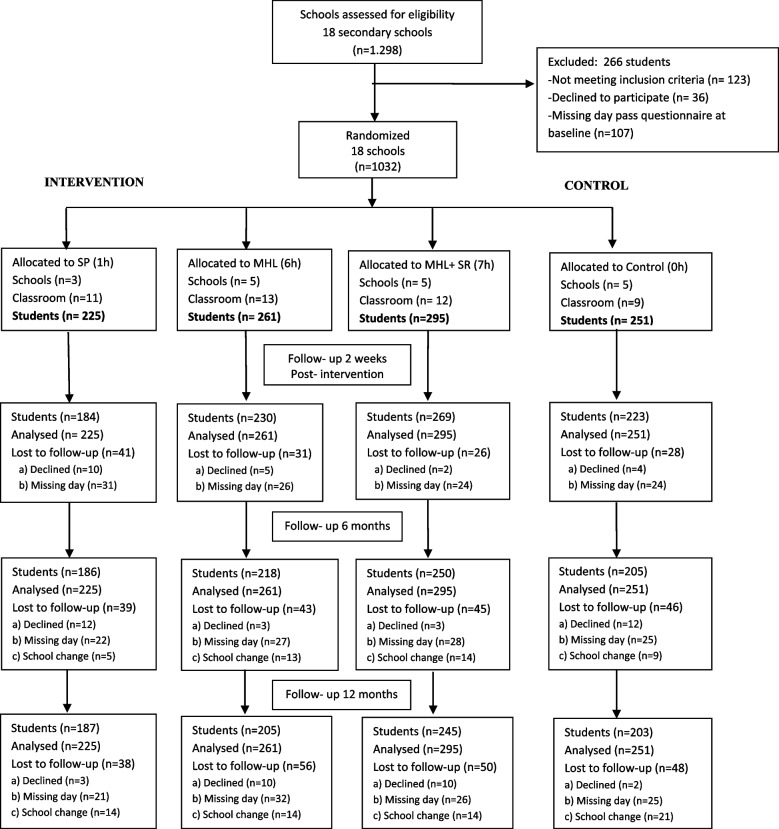
Flow chart of participants *Abbreviations:* MHL Mental Health Literacy Programme; MHL + SR Mental Health Literacy Programme plus Stigma Reduction; SP Sensitivity Programme

A total of 1,298 students were recruited, 1,032 of whom were part of the baseline evaluation and subsequent randomisation; 266 students were excluded due to: 1) Not meeting inclusion criteria (*n* = 123); 2) Declining to participate (*n* = 36); 3) Missing day pass questionnaire at baseline (*n* = 107). The CONSORT diagram is shown in Fig. 1.

Of the 1,032 students who participated in the study, 87.8% (*n* = 906) completed both pre-post questionnaires, 83% (*n* = 859) completed both pre- and six-month follow up and 81.3% (*n* = 840) completed both pre- and 12-month follow up. Comparing the subjects who have complete data (*n* = 726) with those who had an imputed value in any of the evaluations (*n* = 306) significant differences are found in age (*p* < 0.001) and nationality (*p* = 0.019) at baseline. The median age was 14.12 (SD 0.51) and 14.38 (SD 0.66) respectively. There were more Spanish students (*n* = 588; 81%) in the complete data group when compare with the dropout group (*n* = 228; 74.5%). For the rest of the sociodemographic variables, no significant differences were observed. All sociodemographic variables were taken into account in the imputation of missing data.

Of the general sample, 295 (28.6%) received the MHL + SR programme (7 h), 261 (25.3%) received the MHL  programme (6 h), 225 (21.8%) received the SP programme (1 h) and 251 (24.3%) were part of the control group. Table 2 shows the characteristics of the general study population and according to intervention group.

**Table 2:** Descriptive characteristics of the study population

**Variables**	**Category**	**General** **(*n* = 1032)** N (%)	**Control Group** **(*n* = 251)** N (%)	**Intervention Group (1 h)** **(*n* = 225)** N (%)	**Intervention Group (6 h)** **(*n* = 261)** N (%)	**Intervention Group (7 h)** **(*n* = 295)** N (%)	**p**
**Gender**	Women	512 (49.6%)	127 (50.6%)	118 (52.4%)	130 (49.8%)	137 (46.4%)	0.57
	Men	520 (50.4%)	124 (49.4%)	107 (47.6%)	131 (50.2%)	158 (53.6%)	
**Age**	Mean (DE)	14.2 (0.58)	14.2 (0.57)	14.2 (0.58)	14.2 (0.63)	14.2 (0.53)	0.26
**Nationality**	Spanish	816 (79.1%)	194 (77.3%)	156 (69.3%)	225 (86.2%)	241 (81.7%)	** < 0.001**
**School**	Public	922 (89.3%)	225 (89.6%)	225 (100%)	233 (89.3%)	239 (81%)	** < 0.001**
	Private	110 (10.7%)	26 (10.4%)	–-	28 (10.7%)	56 (19%)	
**Psychological Help for a mental health problem**	Yes	288 (28.2%)	70 (28%)	70 (31.4%)	78 (30.1%)	70 (24.1%)	0.25
**Medication for a mental health problem**	Yes	49 (5%)	13 (5.5%)	11 (5.3%)	14 (5.6%)	11 (3.8%)	0.68
**EMHL Test First Part**	Baseline Mean (SD)	7.22 (1.45)	6.94 (1.45)	7.12 (1.53)	7.37 (1.40)	7.41 (1.41)	** < 0.001**
**EMHL Test Second Part**	Baseline Mean(SD)	4.23 (1.14)	4.02 (1.12)	4.14 (1.71)	4.38 (1.08)	4.36(1.15)	** < 0.001**
**CAMI**	Baseline Mean (SD)	27.60 (4.48)	28.1 (4.09)	27.4 (4.73)	27.8 (4.75)	27.2 (4.32)	0.12
**RIBS**	Baseline Mean (SD)	8.83 (3.36)	8.94 (3.42)	8.40 (3.23)	8.79 (3.42)	9.09 (3.34)	0.12
**GHSQ**	Friend	4.92 (1.83)	5.13 (1.70)	4.75 (1.85)	4.88 (1.91)	4.90 (1.83)	0.14
	Parent	5.19 (1.94)	5.11 (1.94)	5.20 (1.88)	5.34 ( 1.91)	5.12 ( 1.99)	0.51
	Teacher	2.78 (1.78)	2.78 (1.98)	2.85 (1.64)	2.70 ( 1.71)	2.80 ( 1.78)	0.81
	Mental health professional	4.47 (2.14)	4.31 (2.22)	4.59 (2.03)	4.71 (2.03)	4.29 (2.22)	0.06
	No one	2.35 (1.96)	2.30 (1.97)	2.44 (1.94)	2.08 (1.78)	2.56 (2.10)	**0.03**

*Abbreviations: CAMI* Scaling Community Attitudes toward the Mentally Ill, *EMHL* EspaiJove Mental Health Literacy test, *GHSQ* General Help-seeking Questionnaire, *RIBS* Reported and Intended Behaviour Scale

^*^ *p* < 0.001, **p < 0.05

The average age of participants was 14.2 (SD 0.58; range 13–16 years old). Significant differences were found between groups in the variable nationality (*p* < 0.001) and type of school (*p* < 0.001). There were more children of foreign nationality (eg Philippines, Pakistan, Bolivia, China, Morocco, etc.) in the control and SP intervention (1 h) groups. These schools were located in areas with more migration in the city. The majority of the schools that participated in the study were public. Of the students, 28.2% (*n* = 288) reported having received psychological help for a mental health problem and 5% (*n* = 49) had been prescribed medication for a mental health problem. Of these, only 19 students reported the prescribed medication (antidepressants (*n* = 3); anxiolytics (*n* = 2); attention deficit hyperactivity disorder (*n* = 13) and antipsychotic (*n* = 1)).

### Mental health literacy

If we use the non-imputed data from the First part of the EMHLT as a reference, we observed a mean change of 0.15 in the MHL + SR group (CI 0.04–0.27; *p* = 0.007) compared to the CG, and of 0.10 (CI 0.00–0.19; *p* = 0.043) compared to the general sample, both being significant. In the Second part of the EMHLT, the non-imputed data did not show significant changes in the MHL + SR intervention compared to the control group and the overall sample (*p* = 0.588 and 0.892 respectively).

Table 3 shows the scores of the First and Second Part of the EspaiJove Mental Health Literacy test (EMHLT) in the different groups throughout the four assessments. According to the imputed data, although a trend of increasing knowledge was found in both parts of the questionnaire in the MHL and MHL + SR groups post-intervention and at 12-month follow-up, in comparison with the SP and the CG, no significant differences were found between groups (SP, MHL and MHL + SR) over time in either of the two parts (*p* = 0.52 and 0.62 respectively).

**Table 3:** Changes in MHL, Stigma and Help-seeking over time

**Variables**	**Category**	**Control group** (*n* = 251)	**Intervention group (1 h) (SP)** (*n* = 225)	**Intervention group (6 h) (MHL)** (*n* = 261)	**Intervention group (7 h) (MHL + SP)** (*n* = 295)	*p*-value*
**MENTAL HEALT LITERACY TEST**						
** EMHL Test First Part** Mean (SD)	**Baseline**	6.94 (1.45)	7.12 (1.53)	7.37 (1.40)	7.41 (1.41)	0.58
	**Post-intervention**	7.23 (1.59)	7.87 (1.76)	8.25 (1.58)	8.31 (1.47)	
	**6 m Follow up**	7.38 (1.60)	7.75 (1.79)	8.08 (1.64)	8.20 (1.57)	
	**12 m Follow up**	7.49 (1.59)	7.70 (1.70)	8.18 (1.63)	8.30 (1.58)	
** EMHL Test Second Part**	**Baseline**	4.02 (1.12)	4.14 (1.71)	4.38 (1.08)	4.36 (1.15)	0.73
	**Post-intervention**	4.12 (1.43)	4.60 (1.54)	5.25 (1.64)	5.15 (1.43)	
	**6 m Follow up**	4.33 (1.60)	4.51 (1.52)	5.08 (1.52)	4.93 (1.57)	
	**12 m Follow up**	4.51 (1.60)	4.46 (1.59)	5.01 (1.61)	4.92 (1.60)	
**STIGMA**						
** CAMI**	**Baseline**	28.1 (4.09)	27.4 (4.73)	27.8 (4.75)	27.2 (4.32)	0.58
	**Post-intervention**	27.5 (4.66)	26.4(5.25)	25.6 (5.57)	25.3 (5.11)	
	**6 m Follow up**	27.1 (5.12)	26.1 (5.55)	25.4 (5.45)	25.1 (5.64)	
	**12 m Follow up**	26.5 (5.35)	26.0 (5.65)	25.4 (5.69)	25.2 (5.51)	
** RIBS**	**Baseline**	8.94 (3.42)	8.40 (3.23)	8.79 (3.42)	9.09 (3.34)	0.77
	**Post-intervention**	8.91 (4.00)	7.97 (3.61)	8.05 (3.58)	8.16 (3.47)	
	**6 m Follow up**	8.55(3.80)	8.23 (3.82)	7.99 (3.66)	8.17 (3.86)	
	**12 m Follow up**	8.20 (3.81)	7.94 (3.80)	7.89 (3.69)	7.92 (3.72)	
**HELP SEEKING**						
** Friend** Mean (SD)	**Baseline**	5.13 (1.70)	4.75 ( 1.85)	4.88 (1.91)	4.90 (1.83)	0.79
	**6 m Follow up**	4.49 (2.27)	4.50 (2.15)	4.52 (2.20)	4.41 (2.18)	
	**12 m Follow up**	4.43 (2.26)	4.39 (2.23)	4.45 (2.27)	4.69 (2.22)	
** Parent**	**Baseline**	5.11 (1.94)	5.20 (1.88)	5.34 ( 1.91)	5.12 ( 1.99)	0.91
	**6 m Follow up**	4.67 (2.21)	4.61 (2.22)	4.80 (2.19)	4.69 (2.21)	
	**12 m Follow up**	4.54(2.24)	4.45 (2.11)	4.69 (2.24)	4.62 (2.11)	
** Teacher**	**Baseline**	2.78 (1.98)	2.85 (1.64)	2.70 ( 1.71)	2.80 ( 1.78)	0.44
	**6 m Follow up**	3.66 (2.18)	3.02 (1.95)	3.22 (2.12)	3.19 (2.10)	
	**12 m Follow up**	3.57 (2.15)	3.07 (2.04)	3.52 (2.21)	3.23 (2.09)	
** Mental health professional**	**Baseline**	4.31 (2.22)	4.59 ( 2.03)	4.71 (2.03)	4.29 (2.22)	0.65
	**6 m Follow up**	3.84 (2.27)	3.90 (2.14)	4.10 (2.18)	3.97 (2.26)	
	**12 m Follow up**	3.88(2.25)	3.82 (2.11)	4.11 (2.25)	3.90 (2.20)	
** No one**	**Baseline**	2.30 (1.97)	2.44 (1.94)	2.08 (1.78)	2.56 (2.10)	0.99
	**6 m Follow up**	3.04 (2.40)	2.72 (2.16)	2.92 (2.28)	2.85(2.25)	
	**12 m Follow up**	3.20 (2.39)	3.20 (2.31)	3.05 (2.43)	2.96 (2.31)	

*Abbreviations: CAMI* Scaling Community Attitudes toward the Mentally Ill, *EMHL* EspaiJove Mental Health Literacy test, *MHL* Mental Health Literacy Programme, *MHL* + *SR* Mental Health Literacy Programme plus Stigma Reduction, *RIBS* Reported and Intended Behaviour Scale, *SP* Sensitivity Programme

^*^p value for interaction time* group adjusted for: gender, nationality, psychological help or medication for a mental health

### Stigma

Table 3 shows the CAMI and RIBS questionnaire scores in the different groups throughout the four assessments. According to the imputed data, no significant differences were found between groups over time in either of the two scales (*p* = 0.61 and 0.90 respectively).

In the CAMI questionnaire, a more marked reduction in the score was found in the MHL and MHL + SR groups post-intervention and at 12-month follow-up, compared to the other two groups (SP and CG). The RIBS questionnaire showed similar results to the CAMI questionnaire, although in this case, MHL + SR group showed a more marked reduction in the score post-intervention and in the 12-month follow-up, compared to the other three groups (MHL, SP and CG).

### Help seeking

Table 3 shows the GHSQ scores in the different groups throughout the three assessments. According to the imputed data, no significant differences were found between groups over time in any of the five subscales (*Friend p* = 0.24; *Parent p* = 0.69; *Teacher p* = 0.31; *Mental Health Professional p* = 0.75 and *No-one p* = 0.45).

### Acceptability and satisfaction

**Fig. 2:**
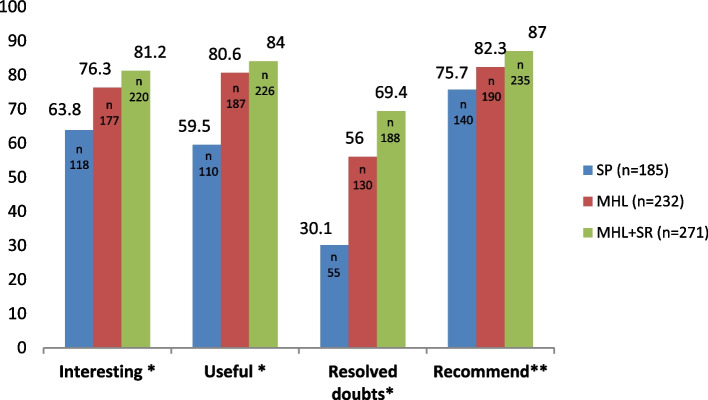
Percentage of acceptability and satisfaction with the different programs *Abbreviations:* MHL Mental Health Literacy; MHL + SR Mental Health Literacy plus Stigma Reduction; SP Sensitivity Programme. * p < 0.001, **p < 0.01

Of the sample of 781 students who participated in any of the three interventions, 688 (88%) completed the satisfaction questionnaire after completing the intervention (SP; *n* = 185) (MHL; *n* = 232) (MHL + SR; *n* = 271). Figure 2 shows the scores of the students who assessed each of the four items (interesting, useful, practical and recommend) with the option “very satisfied” according to the three intervention groups. Statistically significant differences were found in the four items (*p* < 0.005), showing that the MHL + SR intervention was the best rated.

The effect side between groups comparisons in all outcomes (mental health literacy, stigma and help-seeking) was small (< 0.5) over time.

## Discussion

This study reports the short- and long-term results of the universal MHL intervention “EspaiJove.net” designed to increase mental health knowledge and help-seeking behaviours and reduce the stigma associated with mental illness in Spanish school settings. We compared three interventions of different durations (Sensitivity Programme (SP), MHL programme and MHL + SR programme) of the EspaiJove.net programme.

Our results differ from the original hypothesis and this may be related to different factors such as the design of the study and the components of Espaijove.net program (content, duration, instrument used, person who delivers intervention and stigma components).

The results show that all three programmes increase mental health literacy in the short term (post-intervention) and long term (6 and 12 months follow-up), and that such increases are more pronounced with the MHL and MHL + SR interventions than with the SP. However, no significant differences were detected between the different intervention groups and the control group and the effect size was small. Our results are not consistent with RCTs school and community-based interventions included in the systematic review by Seedaket [22], in which statistically significant increase in mental health literacy was observed in one-hour interventions [41] and in longer interventions [42, 43, 18].

Our results may be affected by the content and duration of our program. Maybe the scope of the program was too wide including sessions focused on general aspects of mental health (emotional management, healthy and risk behaviours, social skills and antisocial behaviours), and sessions focused on particularly common mental illness (e.g. depression, anxiety, eating disorders, psychotic disorders) in a six hours program. This duration might have been too short considering the scope of the program. It is worth highlighting that most MHL interventions address general mental health or specific mental disorders, and have a duration between one and twelve hours. Further research should include the study of the effectiveness of our intervention with a longer duration.

There is also a diversity of instruments used to measure the concept of MHL, which were mostly developed by the authors of the programmes. In our case, the EMHL test [31] included all contents of the Espaijove.net, wanting to encompass the broadest definition of MHL. Most studies used instruments to identify specific mental disorders [42, 43, 18] or general mental health topics [44]. Therefore, this heterogeneity of measures makes it difficult to compare results.

Another aspect to be considered in MHL interventions is the professional who carries them out. In most studies, teachers are who carry out the intervention [42, 43, 18] and in the systematic review by Fretian [25] it is shown that knowledge and attitudes on MHL improve when both professionals and teachers delivered the intervention. In our study, they were run by community nurses, who were specialists in mental health, with the knowledge and skills to carry out mental health promotion programmes in community environments, such as schools. Our aim was to bring mental health professionals closer to schools, and thus reduce negative beliefs towards mental health services and professionals, identified as a barrier when seeking help in Aguirre’s study [6]. Maybe the three interventions would have worked better if carried out jointly with teachers, as the existing literature shows.

These aspects were discussed in the systematic review by Mansfield et al. [45], where the authors suggest a better understanding of what MHL means for this population, as well as the need to develop reliable, valid and feasible measurements. They also suggest moving from a definition of MHL focused on training the population in health issues with a biomedical orientation based on the contents of the Diagnostic and Statistical Manual of Mental Disorders [46] towards one that takes into account aspects of positive psychology “the self-generated and acquired knowledge with which people negotiate their mental health” which includes topics such as resilience, salutogenesis and mindfulness [47]. Another proposed approach is to improve the mental health of this population based on behaviour change, which entails shifting the current emphasis on “*mental health literacy*” towards “*mental health action*”, defined as “*action that individuals or groups take to benefit their own mental health or that of others*”[48].

In relation to stigma, the MHL and MHL + SR interventions reduced attitudes in the short and long term, compared to the SP and CG interventions, but these reductions were not statistically significant and the effect size between groups was small. We found that the MHL + SR intervention, where stigma is addressed through direct contact with a person who has suffered from a mental disorder, obtained similar results to the MHL intervention. Our results are consistent with the study by Chisholm [18], which showed that the combination of an educational intervention and first-person experience did not help to reduce stigma. Interventions which used first-person experience, either face-to-face or by means of digital stories, show an increase in mental health knowledge, but inconsistent results in relation to stigma [41, 43, 18, 44]. Most of community-based interventions differ in the format of imparting the stigma component (education, education plus contact condition, digital stories) as shown by the review of Nobre [49]. There are controversies regarding the duration (hours/sessions), how many people have to participate (individual or a group) or whether educational interventions are more effective in reducing stigma compared to interventions that included first-person contact [25, 24]. The meta-analysis by Fretian [25] showed that contact interventions are not more effective in reducing stigma than education interventions, and the results suggest to identify which intervention components are more effective.

Most studies used validated instruments to assess stigma that have been validated in young population in recent years [50, 34]. In our case, we used a validated instrument in such a population, the fact that we only included one dimensions of the instrument may have interfered in the study of the effects of the intervention. On the other hand, we found that the young people who participated in the study showed low stigmatising behaviours at baseline towards people who suffer from a mental disorder. Others studies in similar populations have showed similar results [18, 51, 34]. Therefore, the fact that no significant differences were found between the different interventions could be due to the ceiling effect of the instruments used.

Moreover, we found the MHL + SR intervention to be the most highly rated by the young people, in terms of finding it useful and interesting, that it resolved their doubts, and that they would recommend it to other young people. The positive rating shows the importance of the first-person experience as a factor to be included in mental health literacy programmes in the school environment, perhaps not aimed at reducing stigmatising attitudes, but certainly for taking the subject of mental health into the classroom.

There was no significant improvement found between the different interventions in the seeking of help for a mental health problem. The results showed that they would seek help initially from their parents, followed by a friend, a healthcare professional, a teacher and, lastly, from nobody; these results were maintained at the 12-month follow-up. The family was identified as a source when seeking for help, although there was still a proportion of adolescents who would not seek help from anybody.

These results are in line with other similar studies [18], although it is difficult to compare them owing to the variability in the instruments used. Our results are similar to those found in the study by Olivari [52], in which young people preferred to seek help for a mental health problem from informal sources, such as friends and parents, and not formal sources, such as psychologists, psychiatrists, doctors and teachers/lecturers. These data suggest that families should also be included in programmes in order to help them detect early signs of young people needing mental healthcare, and thus improve their referral to specialised services.

Our results may have been influenced by the type of study design. We compared four different interventions at the same time; while most of previous studies compare only two. This methodological issue may have affected the statistical power required to detect differences between interventions groups and, this may explain why our results are not consistent with RCTs school and community-based interventions included in the systematic review by Seedaket [22]. We also observed that the students who were part of the control group also improved their knowledge and decreased their attitudes of stigma in the short and long term. Therefore, we observe that it is a population in continuous change, an aspect to be considered in future interventions.

Our study has a number of limitations. Firstly, the selection of the study sample was limited to Barcelona city which may affect generalisation of the results to the rest of the Spanish population. Although initially a sample calculation of 408 students was made, ultimately 1,032 students from 18 schools participated. The participating schools came from all the neighbourhoods of Barcelona, representing the diversity of its educational system, which may increase the chances to generalize the results. Secondly, the loss of subjects to follow-up. Loss to follow-up accounted for around 12% at post-intervention, 16.7% at six months and 18.6% at 12 months. Considering these figures, there may be a possible decrease in statistical power. Even so, these percentages are similar to those of other studies carried out in the same setting. Thirdly, this study contains many outcomes, and no correction for multiple testing has been used to control for the false positive rate. However, most of these outcomes are related to each other. Fourth, the instruments used to assess stigma, may not be sensitive to assessing changes may be to ceiling effect. Although the instruments used have been validated in young population in recent years.

Our study has a number of strengths, including the extensive external validity of the study despite it being a cRCT. The study was conducted as close as possible to the actual conditions of the school setting, with the objective of being implemented in other secondary schools within Barcelona city in Catalonia in the future. The sample size of 1032 students was higher than initially calculated (408 students). This initial calculation was based on the mental health knowledge variable, but more outcomes variables have been included in the study. Increasing the sample size allows us more precision of other variables and allows us to analyze results in different clusters. It is also the first study to evaluate the short- and long-term effectiveness of a universal MHL programme in Spain.

Further research of universal MHL interventions (> 6 h) with cRCT design carried out by nurses and teachers together in a school setting, which share the same MHL concept, duration and objective measurement tools is needed to confirm these findings.

Future interventions should take into account the concept of MHL used to define the different MHL interventions, the duration thereof, and the use of specific, validated questionnaires for each of the programme components (mental health literacy, knowledge of mental disorders and treatments, stigma and seeking help). Moreover, first-person interventions should focus on increasing knowledge and reducing stigma in mental health. Simultaneous MHL interventions with families should also be considered, since adults in the family are identified as points of reference and as those who can facilitate the referral of young people seeking help to mental health professionals.

## Conclusions

The three interventions of the EspaiJove.net programme (SP, MHL and MHL + SR) seem not to be effective in terms MHL, Stigma and help-seeking behaviours in the short (post-intervention) and long term (6 and 12 months follow up). The contact with a person who has experimented mental illness first-hand did not reduce stigma attitudes, but this intervention was the most highly rated by the young people. Further research should deal with the heterogeneity of MHL interventions (concept, duration and measures) and identify which components of stigma interventions are effective.

## Supplementary Information

**Additional file 1:** **Supplementary material 1.** Description Espaijove.net program.

**Additional file 2:** **Supplementary material 2.** Number of subjects contributed by each school.

**Additional file 3:** **Supplementary material 3.** Changes in MHL, Stigma and Help-seeking over time.

**Additional file 4:** **Supplementary material 4.** Complete models with imputed data.

**Additional file 5:** **Supplementary material 5.** Effect size in MHL, Stigma and Help-seeking according to treatment groups over time.

## Data Availability

The Prezi format presentations for the 6 modules of the EspaiJove.net programme are accessible via the following website: www.espaijove.net. The datasets generated and/or analysed during the current study are not publicly available due to privacy concerns but are available from the corresponding author on reasonable request.

## Abbreviations

CAMI: Community Attitudes toward the Mentally Ill
cRCT: Clustered Randomised Controlled Trial
EMHLT: EspaiJove.net Mental Health Literacy test
GHSQ: General Help-Seeking Questionnaire
MHL: Mental Health Literacy
MHL + SR: Mental Health Literacy plus Stigma Reduction
RIBS: Reported and Intended Behaviour Scale
SP: Sensitivity Programme
