# Supplementary material for: The effectiveness of a “EspaiJove.net”- a school-based intervention programme in increasing mental health knowledge, help seeking and reducing stigma attitudes in the adolescent population: a cluster randomised controlled trial
Source: BMC Public Health. 2022 Dec 24;22:2425. doi: 10.1186/s12889-022-14558-y (PMC9789578; doi:10.1186/s12889-022-14558-y)
Supplement: Supplementary file 1 — Additional file 1: Supplementary material 1. Description Espaijove.net program. [file 12889_2022_14558_MOESM1_ESM.docx]

**Supplementary material 1:** Description Espaijove.net program

***Description Espaijove.net program***

The *EspaiJove.net: a space for mental health* (EspaiJove.net) programme was developed by the Associación Centre Higiene Mental Les Corts (CHMLC) and it has been carried out since 2012 in the city of Barcelona (Spain). It is a universal Mental Health Literacy (MHL) programme which aims to promote mental health, prevent mental disorders, facilitate help-seeking behaviours and eradicate related stigma among secondary school students (between 11 and 18 years old). The programme integrates a multi-modal intervention that combines workshops and training activities with the use of Information and Communication Technology (ICT) such as the website www.espaijove.net that includes online consultation.

The *EspaiJove.net program* consists in disseminating information on mental health and carrying out training activities with young people in schools. The project has been designed and is delivered by professionals who are specialised in community mental health (i.e., nurses, psychologist and psychiatrists) and who work in community mental health centers. They carry out the training activities in educational centres. The programme includes education on several key aspects of mental health such as to: mental health wellbeing, help-seeking behaviours on their own, raise awareness of the consequences of risky behaviours, identify mental disorders and when/where to seek treatment, so as to eventually prevent and detect mental health-related problems early. Additionally, a person who has experienced mental illness first-hand will speak about his/her personal life experience with the students so as to aim to reduce any related stigma.

The program began in 2007, coinciding with the implementation of the "Salut i Escola Program" in the districts of Les Corts and Sarrià Sant Gervasi in Barcelona city (Departament de Salut generalitat de Catalunya, 2010). A review of the mental health literacy programs in children and youth that were being carried out internationally were carried out, which could guide de design of our program (Casañas, 2017).

Most of the mental health literacy programs that had shown to be effective were from Anglo-Saxon countries with a culture and level of training different to those of our context. So, we valued the importance of adapting these programs to our context needs. Our intervention is based in the following mental health literacy programs: MindMatters (Wyn 2000), Headspace (Muir, 2009) and Youthspace (Birchwood, 2013).

During 2008-2009, a group of professionals from the CHMLC eleborated the following educational materials: 14 fact sheets on mental health issues aimed at the young population (11 and 18 years old) and 1 manual on mental health aimed at professionals in the health and educational settings.

The fact sheets, which deal with mental disorders, are based on a basic scheme with the following sections: 1.What is it? (Information about the disorder); 2. How do I feel? (Warning signs and risk factors expressed in the first person); 3. How is it presented? (Description of evolutionary normality and the appearance of symptoms); 4. What can I do? (Prevention); 5.How to ask for help? (Contact available resources).

During the years 2011-2012, the educational materials were updated and adapted, increasing the number of information sheets (27 fact sheets) and two specific training manuals: 1)a manual for professionals in the educational, social and community fields; and 2) another manual for professionals in the health field) (Casale 2012a and Casale 2021b) .

The updated fact sheets include: 1) Espaijove.net Program; 2) Adolescence; 3) Healthy behaviors in Mental Health; 4) Risk behaviors in Mental Health; 5) Concept of Mental Health; 6) Our emotions; 7) Duel; 8) Stress and anxiety; 9) Concept of Mental Disorder; 10) Social skills; 11) Bullying; 12) Anxiety; 13) Depression; 14) Self-harm; 15) Suicide; 16) Attention deficit disorder (ADD); 17) Obsessive Compulsive Disorder; 18) Eating disorders (EDs); 19) Borderline Personality Disorder (BPD); 20) Substance Abuse Disorders; 21) Psychotic Disorder; 22) Schizophrenia; 23) Bipolar disorder; 24) Ciberbyllying; 25) Multidisciplinary mental health team; 26) Community service network and 27) Stigma.

Design and operation of the project website (www.espaijove.net). The website contains information related to the physical and mental health of young people, an open consultation on these issues, and social media such as Facebook and Twitter for young people (@EspaiJoveNet) and for professionals (@EspaiJovenetPro).

In the last seven academic courses (2012-2019) workshops have been provided to 24,118 secondary schools students (20 schools), of whom 11,678 completed a post-workshop satisfaction questionnaire. The results show that the program is interesting, useful, has resolved students’ doubts and is recommendable to other students (Casañas, 2020).

**References**

- Birchwood M, Singh SP. Mental health services for young people: matching the service to the need. Br J Psychiatry Suppl. 2013 Jan;54:s1-2. doi: 10.1192/bjp.bp.112.119149. PMID: 23288494.
- Casalé D, Castells G, Pujol A, Gil JJ, Casañas R, Lalucat-Jo Ll. Manual de salud mental para profesionales del ámbito sanitario. Programa de promoció i prevenció de la salut mental «Espai Jove» Les Corts Centre d’Higiene Mental, 2012. Barcelona. http://www.espaijove.net/continguts/MANUAL_SM_SANITARIOS_CAST.pdf
- Casalé D, Castells G, Pujol A, Gil JJ, Casañas R, Lalucat-Jo Ll. Manual de salud mental para profesionales del ámbito educativo en contacto con adolescentes. Programa de promoció i prevenció de la salut mental «Espai Jove» Les Corts Centre d’Higiene Mental, 2012 Barcelona.

http://www.espaijove.net/continguts/MANUAL_SM_EDUCADORES_CAST.pdf

- Casañas R, Gil JJ, Castells G, Pujol A, Torres M, Lalucat-Jo LL. Evaluación de un programa de alfabetización en salud mental para adolescentes. Rev Psiquiatr Salud Ment. 2017;30:105–14.
- Casañas R, Mas-Expósito L, Teixidó M, Lalucat-Jo L. Programas de alfabetización para la promoción de la salud mental en el ámbito escolar Informe SESPAS 2020. (Literacy programs for the promotion of mental health in the school setting. SESPAS Report 2020). Gaceta Sanitaria Volume 34, Supplement 1, 39-47. https://doi.org/10.1016/j.gaceta.2020.06.010
- Departamnet de salut de la Generalitat de Catalunya. Programa Salut i Escola (PSiE) al Pla Salut, Escola i Comunitat (PSEC). Pla transversal per a la promoció i l’educació de la salut en infants i joves (2010). Barcelona. https://repositori.educacio.gencat.cat/handle/20.500.12694/1266?locale-attribute=ca
- Espaijove.net. Fact sheets

http://espaijove.net/continguts/FICHAS_ADOLESCENTES_CAST.pdf

- Muir K, Powell A, Patulny R, Flaxman S, McDermott S, Oprea I, et al. Independent Evaluation of headspace: the National Youth Mental Health Foundation. Social Policy Research Centre, University of New South Wales, 2009. Sydney, Australia.
- Wyn J, Cahill H, Holdsworth R, Rowling L, Carson S. MindMatters, a whole-school approach promoting mental health and wellbeing. Aust N Z J Psychiatry. 2000 Aug;34(4):594-601. doi: 10.1080/j.1440-1614.2000.00748.x. PMID: 10954390.
