# Supplementary material for: The effectiveness of a “EspaiJove.net”- a school-based intervention programme in increasing mental health knowledge, help seeking and reducing stigma attitudes in the adolescent population: a cluster randomised controlled trial
Source: BMC Public Health. 2022 Dec 24;22:2425. doi: 10.1186/s12889-022-14558-y (PMC9789578; doi:10.1186/s12889-022-14558-y)
Supplement: Supplementary file 2 — Additional file 2: Supplementary material 2. Number of subjects contributed by each school. [file 12889_2022_14558_MOESM2_ESM.docx]

**Supplementary material 2:** Number of subjects contributed by each school

| **School** | **Control group**  (n=251) | **Intervention group (1h)**  **SP**  (n=225) | **Intervention group (6h)**  **MHL**  (n=261) | **Intervention group (7h)**  **MHL+SP**  (n=295) | **Overall**  (n=1032) |
| --- | --- | --- | --- | --- | --- |
| **1. School A** | 0 | 0 | 94 | 0 | 94 |
| **2. School B** | 54 | 0 | 0 | 0 | 54 |
| **3. School C** | 60 | 0 | 0 | 0 | 60 |
| **4. School D** | 0 | 0 | 28 | 0 | 28 |
| **5. School E** | 0 | 0 | 0 | 56 | 56 |
| **6. School F** | 0 | 0 | 0 | 45 | 45 |
| **7. School G** | 0 | 60 | 0 | 0 | 60 |
| **8. School H** | 0 | 67 | 0 | 0 | 67 |
| **9. School I** | 47 | 0 | 0 | 0 | 47 |
| **10. School J** | 64 | 0 | 0 | 0 | 64 |
| **11. School K** | 0 | 0 | 0 | 63 | 63 |
| **12. School L** | 0 | 0 | 0 | 27 | 27 |
| **13. School M** | 0 | 98 | 0 | 0 | 98 |
| **14. School N** | 0 | 0 | 9 | 0 | 9 |
| **15. School O** | 0 | 0 | 80 | 0 | 80 |
| **16. School P** | 0 | 0 | 50 | 0 | 50 |
| **17. School Q** | 26 | 0 | 0 | 0 | 26 |
| **18. School R** | 0 | 0 | 0 | 104 | 104 |

***Abbreviations:*** **MHL** Mental Health Literacy Programme; **MHL+SR** Mental Health Literacy Programme plus Stigma Reduction; **SP** Sensitivity Programme.
