# Supplementary material for: The effectiveness of a “EspaiJove.net”- a school-based intervention programme in increasing mental health knowledge, help seeking and reducing stigma attitudes in the adolescent population: a cluster randomised controlled trial
Source: BMC Public Health. 2022 Dec 24;22:2425. doi: 10.1186/s12889-022-14558-y (PMC9789578; doi:10.1186/s12889-022-14558-y)
Supplement: Supplementary file 3 — Additional file 3: Supplementary material 3. Changes in MHL, Stigma and Help-seeking over time. [file 12889_2022_14558_MOESM3_ESM.docx]

**Supplementary material 3:** Changes in MHL, Stigma and Help-seeking over time

| **Variables** | **Category** | **Control group**  (n=251) | **Intervention group (1h)**  **(SP)**  (n=225) | **Intervention group (6h)**  **(MHL)**  (n=261) | **Intervention group (7h)**  **(MHL+SP)**  (n=295) | **p-value** | **p-value*** |
| --- | --- | --- | --- | --- | --- | --- | --- |
| **MENTAL HEALT LITERACY TEST** | | | | | | | |
| **EMHL Test**  **First Part**  Mean (SD) | **Baseline** | 6.94 (1.45) | 7.12 (1.53) | 7.37 (1.40) | 7.41 (1.41) | 0.52 | 0.58 |
|  | **Post-intervention** | 7.23 (1.59) | 7.87 (1.76) | 8.25 (1.58) | 8.31 (1.47) |  |  |
|  | **6 m Follow up** | 7.38 (1.60) | 7.75 (1.79) | 8.08 (1.64) | 8.20 (1.57) |  |  |
|  | **12 m Follow up** | 7.49 (1.59) | 7.70 (1.70) | 8.18 (1.63) | 8.30 (1.58) |  |  |
| **EMHL Test**  **Second Part** | **Baseline** | 4.02 (1.12) | 4.14 (1.71) | 4.38 (1.08) | 4.36 (1.15) | 0.62 | 0.73 |
|  | **Post-intervention** | 4.12 (1.43) | 4.60 (1.54) | 5.25 (1.64) | 5.15 (1.43) |  |  |
|  | **6 m Follow up** | 4.33 (1.60) | 4.51 (1.52) | 5.08 (1.52) | 4.93 (1.57) |  |  |
|  | **12 m Follow up** | 4.51 (1.60) | 4.46 (1.59) | 5.01 (1.61) | 4.92 (1.60) |  |  |
| **STIGMA** | | | | | | | |
| **CAMI** | **Baseline** | 28.1 (4.09) | 27.4 (4.73) | 27.8 (4.75) | 27.2 (4.32) | 0.61 | 0.58 |
|  | **Post-intervention** | 27.5 (4.66) | 26.4(5.25) | 25.6 (5.57) | 25.3 (5.11) |  |  |
|  | **6 m Follow up** | 27.1 (5.12) | 26.1 (5.55) | 25.4 (5.45) | 25.1 (5.64) |  |  |
|  | **12 m Follow up** | 26.5 (5.35) | 26.0 (5.65) | 25.4 (5.69) | 25.2 (5.51) |  |  |
| **RIBS** | **Baseline** | 8.94 (3.42) | 8.40 (3.23) | 8.79 (3.42) | 9.09 (3.34) | 0.98 | 0.77 |
|  | **Post-intervention** | 8.91 (4.00) | 7.97 (3.61) | 8.05 (3.58) | 8.16 (3.47) |  |  |
|  | **6 m Follow up** | 8.55(3.80) | 8.23 (3.82) | 7.99 (3.66) | 8.17 (3.86) |  |  |
|  | **12 m Follow up** | 8.20 (3.81) | 7.94 (3.80) | 7.89 (3.69) | 7.92 (3.72) |  |  |
| **HELP SEEKING** | | | | | | | |
| **Friend**  Mean (SD) | **Baseline** | 5.13 (1.70) | 4.75 ( 1.85) | 4.88 (1.91) | 4.90 (1.83) | 0.24 | 0.79 |
|  | **6 m Follow up** | 4.49 (2.27) | 4.50 (2.15) | 4.52 (2.20) | 4.41 (2.18) |  |  |
|  | **12 m Follow up** | 4.43 (2.26) | 4.39 (2.23) | 4.45 (2.27) | 4.69 (2.22) |  |  |
| **Parent** | **Baseline** | 5.11 (1.94) | 5.20 (1.88) | 5.34 ( 1.91) | 5.12 ( 1.99) | 0.69 | 0.91 |
|  | **6 m Follow up** | 4.67 (2.21) | 4.61 (2.22) | 4.80 (2.19) | 4.69 (2.21) |  |  |
|  | **12 m Follow up** | 4.54(2.24) | 4.45 (2.11) | 4.69 (2.24) | 4.62 (2.11) |  |  |
| **Teacher** | **Baseline** | 2.78 (1.98) | 2.85 (1.64) | 2.70 ( 1.71) | 2.80 ( 1.78) | 0.31 | 0.44 |
|  | **6 m Follow up** | 3.66 (2.18) | 3.02 (1.95) | 3.22 (2.12) | 3.19 (2.10) |  |  |
|  | **12 m Follow up** | 3.57 (2.15) | 3.07 (2.04) | 3.52 (2.21) | 3.23 (2.09) |  |  |
| **Mental health professional** | **Baseline** | 4.31 (2.22) | 4.59 ( 2.03) | 4.71 (2.03) | 4.29 (2.22) | 0.75 | 0.65 |
|  | **6 m Follow up** | 3.84 (2.27) | 3.90 (2.14) | 4.10 (2.18) | 3.97 (2.26) |  |  |
|  | **12 m Follow up** | 3.88(2.25) | 3.82 (2.11) | 4.11 (2.25) | 3.90 (2.20) |  |  |
| **No one** | **Baseline** | 2.30 (1.97) | 2.44 (1.94) | 2.08 (1.78) | 2.56 (2.10) | 0.45 | 0.99 |
|  | **6 m Follow up** | 3.04 (2.40) | 2.72 (2.16) | 2.92 (2.28) | 2.85(2.25) |  |  |
|  | **12 m Follow up** | 3.20 (2.39) | 3.20 (2.31) | 3.05 (2.43) | 2.96 (2.31) |  |  |

***Abbreviations:*** **CAMI** Scaling Community Attitudes toward the Mentally Ill; **EMHL** EspaiJove Mental Health Literacy Test; **MHL** Mental Health Literacy Programme; **MHL+SR** Mental Health Literacy Programme plus Stigma Reduction; **RIBS** Reported and Intended Behaviour Scale; **SP** Sensitivity Programme.

*p value for interaction time* group adjusted for: gender, nationality, psychological help or medication for a mental health.
