# Supplementary material for: The effectiveness of a “EspaiJove.net”- a school-based intervention programme in increasing mental health knowledge, help seeking and reducing stigma attitudes in the adolescent population: a cluster randomised controlled trial
Source: BMC Public Health. 2022 Dec 24;22:2425. doi: 10.1186/s12889-022-14558-y (PMC9789578; doi:10.1186/s12889-022-14558-y)
Supplement: Supplementary file 4 — Additional file 4: Supplementary material 4. Complete models with imputed data. [file 12889_2022_14558_MOESM4_ESM.docx]

**Supplementary material 4:** Complete models with imputed data

| **Outcome: EMHL Test Firt Part** | | | | | |
| --- | --- | --- | --- | --- | --- |
| **term** | **estimate** | **std.error** | **statistic** | **df** | **p.value** |
| (Intercept) | 7.039 | 0.515 | 13.668 | 36.850 | 0.000 |
| Intervention_ControlGrup Intervention | 0.495 | 0.236 | 2.094 | 184.482 | 0.038 |
| Visit_number | 0.200 | 0.226 | 0.885 | 18.282 | 0.387 |
| sexMale | -0.512 | 0.057 | -8.967 | 70.307 | 0.000 |
| Nationality_Foreign | -0.262 | 0.177 | -1.482 | 11.345 | 0.166 |
| HelpSeeking_psychologicalYes | 0.195 | 0.383 | 0.509 | 8.615 | 0.624 |
| HelpSeeking_medicationYes | -0.393 | 0.426 | -0.923 | 9.061 | 0.380 |
| Intervention_ControlGrup Intervention_visit_number | 0.049 | 0.088 | 0.556 | 17.596 | **0.585** |

*Intraclass correlation coefficient (ICC) 0.12*

| **Outcome: EMHL Test Second Part** | | | | | |
| --- | --- | --- | --- | --- | --- |
| **term** | **estimate** | **std.error** | **statistic** | **df** | **p.value** |
| (Intercept) | 4.208 | 0.615 | 6.843 | 40.579 | 0.000 |
| Intervention_ControlGrup Intervention | 0.495 | 0.238 | 2.075 | 600.485 | 0.038 |
| Visit_number | 0.036 | 0.233 | 0.153 | 28.151 | 0.880 |
| sexMale | -0.272 | 0.055 | -4.902 | 71.548 | 0.000 |
| Nationality_Foreign | -0.346 | 0.162 | -2.132 | 11.755 | 0.055 |
| HelpSeeking_psychologicalYes | -0.016 | 0.571 | -0.028 | 7.544 | 0.978 |
| HelpSeeking_medicationYes | -0.092 | 0.506 | -0.182 | 8.615 | 0.860 |
| Intervention_ControlGrup Intervention_visit_number | -0.020 | 0.060 | -0.336 | 52.186 | **0.738** |

*ICC 0.13*

| **Outcome: CAMI** | | | | | |
| --- | --- | --- | --- | --- | --- |
| **term** | **estimate** | **std.error** | **statistic** | **df** | **p.value** |
| (Intercept) | 27.680 | 1.993 | 13.887 | 22.450 | 0.000 |
| Intervention_ControlGrup Intervention | -0.902 | 0.702 | -1.284 | 252.719 | 0.200 |
| Visit_number | -0.530 | 0.744 | -0.713 | 19.166 | 0.485 |
| sexMale | 1.617 | 0.200 | 8.064 | 62.253 | 0.000 |
| Nationality_Foreign | 1.025 | 0.259 | 3.965 | 74.970 | 0.000 |
| HelpSeeking_psychologicalYes | -0.392 | 1.775 | -0.221 | 7.906 | 0.831 |
| HelpSeeking_medicationYes | 0.479 | 1.907 | 0.251 | 8.472 | 0.808 |
| Intervention_ControlGrup Intervention_visit_number | -0.107 | 0.197 | -0.542 | 92.138 | **0.589** |

*ICC 0.06*

| **Outcome: RIBS** | | | | | |
| --- | --- | --- | --- | --- | --- |
| **term** | **estimate** | **std.error** | **statistic** | **df** | **p.value** |
| (Intercept) | 8.980 | 0.699 | 12.840 | 28.774 | 0.000 |
| Intervention_ControlGrup Intervention | -0.419 | 0.413 | -1.014 | 1031.812 | 0.311 |
| Visit_number | -0.325 | 0.315 | -1.032 | 14.877 | 0.318 |
| sexMale | 0.843 | 0.126 | 6.703 | 141.689 | 0.000 |
| Nationality_Foreign | 0.927 | 0.147 | 6.322 | 2499.715 | 0.000 |
| HelpSeeking_psychologicalYes | -0.457 | 0.760 | -0.601 | 8.994 | 0.562 |
| HelpSeeking_medicationYes | -0.055 | 0.800 | -0.069 | 9.674 | 0.946 |
| Intervention_ControlGrup Intervention_visit_number | 0.035 | 0.123 | 0.287 | 343.365 | **0.774** |

*ICC 0.05*

| **Outcome: Help seeking Friend** | | | | | |
| --- | --- | --- | --- | --- | --- |
| **term** | **estimate** | **std.error** | **statistic** | **df** | **p.value** |
| (Intercept) | 5.334 | 1.418 | 3.762 | 98.686 | 0.000 |
| Intervention_ControlGrup Intervention | -0.078 | 0.670 | -0.117 | 10.410 | 0.909 |
| Visit_number | 0.041 | 0.435 | 0.095 | 3739.725 | 0.925 |
| sexMale | -0.361 | 0.080 | -4.502 | 26.529 | 0.000 |
| Nationality_Foreign | -0.329 | 0.359 | -0.915 | 9.103 | 0.384 |
| HelpSeeking_psychologicalYes | -0.397 | 0.705 | -0.563 | 7.320 | 0.590 |
| HelpSeeking_medicationYes | 0.028 | 0.878 | 0.032 | 7.540 | 0.975 |
| Intervention_ControlGrup Intervention_visit_number | 0.030 | 0.116 | 0.259 | 14.400 | **0.799** |

*ICC 0.12*

| **Outcome: Help seeking Parent** | | | | | |
| --- | --- | --- | --- | --- | --- |
| **term** | **estimate** | **std.error** | **statistic** | **df** | **p.value** |
| (Intercept) | 5.007 | 1.573 | 3.182 | 107.941 | 0.002 |
| Intervention_ControlGrup Intervention | -0.172 | 0.569 | -0.302 | 10.960 | 0.768 |
| Visit_number | 0.080 | 0.498 | 0.160 | 1886.679 | 0.873 |
| sexMale | 0.212 | 0.063 | 3.343 | 96.574 | 0.001 |
| Nationality_Foreign | -0.068 | 0.370 | -0.183 | 9.078 | 0.859 |
| HelpSeeking_psychologicalYes | -0.533 | 0.510 | -1.046 | 8.290 | 0.325 |
| HelpSeeking_medicationYes | -0.368 | 0.717 | -0.514 | 8.165 | 0.621 |
| Intervention_ControlGrup Intervention_visit_number | -0.009 | 0.079 | -0.112 | 33.732 | **0.912** |

*ICC 0.03*

| **Outcome: Help seeking Teacher** | | | | | |
| --- | --- | --- | --- | --- | --- |
| **term** | **estimate** | **std.error** | **statistic** | **df** | **p.value** |
| (Intercept) | 3.327 | 1.613 | 2.063 | 152.509 | 0.041 |
| Intervention_ControlGrup Intervention | -0.025 | 0.320 | -0.079 | 55.976 | 0.938 |
| Visit_number | 0.001 | 0.526 | 0.002 | 1631.368 | 0.999 |
| sexMale | 0.134 | 0.072 | 1.861 | 35.467 | 0.071 |
| Nationality_Foreign | 0.103 | 0.262 | 0.391 | 9.955 | 0.704 |
| HelpSeeking_psychologicalYes | -0.153 | 0.478 | -0.319 | 8.330 | 0.757 |
| HelpSeeking_medicationYes | 0.017 | 0.380 | 0.044 | 9.589 | 0.966 |
| Intervention_ControlGrup Intervention_visit_number | -0.047 | 0.061 | -0.767 | 129.469 | **0.444** |

*ICC 0.11*

| **Outcome: Help seeking Mental Health Professional** | | | | | |
| --- | --- | --- | --- | --- | --- |
| **term** | **estimate** | **std.error** | **statistic** | **df** | **p.value** |
| (Intercept) | 4.089 | 1.262 | 3.239 | 103.365 | 0.002 |
| Intervention_ControlGrup Intervention | 0.241 | 0.314 | 0.767 | 23.437 | 0.451 |
| Visit_number | 0.045 | 0.391 | 0.114 | 3518.188 | 0.909 |
| sexMale | 0.386 | 0.132 | 2.921 | 14.541 | 0.011 |
| Nationality_Foreign | -0.130 | 0.500 | -0.260 | 8.669 | 0.801 |
| HelpSeeking_psychologicalYes | 0.002 | 0.536 | 0.004 | 8.532 | 0.997 |
| HelpSeeking_medicationYes | -0.566 | 0.556 | -1.019 | 9.171 | 0.334 |
| Intervention_ControlGrup Intervention_visit_number | -0.035 | 0.078 | -0.445 | 86.456 | **0.657** |

*ICC 0.01*

| Outcome: **Help seeking No one** | | | | | |
| --- | --- | --- | --- | --- | --- |
| **term** | **estimate** | **std.error** | **statistic** | **df** | **p.value** |
| (Intercept) | 2.878 | 2.033 | 1.416 | 116.215 | 0.160 |
| Intervention_ControlGrup Intervention | -0.135 | 0.495 | -0.273 | 13.087 | 0.789 |
| Visit_number | 0.034 | 0.635 | 0.053 | 3852.055 | 0.958 |
| sexMale | -0.152 | 0.098 | -1.548 | 20.846 | 0.137 |
| Nationality_Foreign | 0.266 | 0.236 | 1.129 | 10.946 | 0.283 |
| HelpSeeking_psychologicalYes | 0.394 | 0.659 | 0.598 | 7.871 | 0.567 |
| HelpSeeking_medicationYes | 0.098 | 0.576 | 0.170 | 8.903 | 0.869 |
| Intervention_ControlGrup Intervention_visit_number | -0.001 | 0.104 | -0.013 | 19.813 | **0.990** |

*ICC 0.03*
