# Supplementary material for: The effectiveness of a “EspaiJove.net”- a school-based intervention programme in increasing mental health knowledge, help seeking and reducing stigma attitudes in the adolescent population: a cluster randomised controlled trial
Source: BMC Public Health. 2022 Dec 24;22:2425. doi: 10.1186/s12889-022-14558-y (PMC9789578; doi:10.1186/s12889-022-14558-y)
Supplement: Supplementary file 5 — Additional file 5: Supplementary material 5. Effect size in MHL, Stigma and Help-seeking according to treatment groups over time. [file 12889_2022_14558_MOESM5_ESM.docx]

**Supplementary material 5:** Effect size in MHL, Stigma and Help-seeking according to treatment groups over time

| **Variables** | **Category** | **Intervention group (1h) (SP)**  (n=225)  Cohens d* (CI) | **Intervention group (6h) (MHL)**  (n=261)  Cohens d (CI) | **Intervention group (7h) (MHL+SP)**  (n=295)  Cohens d (CI) |
| --- | --- | --- | --- | --- |
| **MENTAL HELATH LITERACY** | | | | |
| **EMHL Test**  **First Part** | **Post-intervention** | -0.352 (-0.53 to -0.17) | -0.309 (-0.48 to -0.13) | -0.218 (-0.39 to -0.05) |
|  | **6 m Follow up** | -0.207 (-0.39 to -0.03) | -0.190 (-0.36 to -0.02) | -0.121 (-0.29 to 0.05) |
|  | **12 m Follow up** | -0.177 (-0.36 to 0.00) | -0.212 (-0.38 to -0.04) | -0.152 (-0.32 to 0.02) |
| **EMHL Test**  **Second Part** | **Post-intervention** | -0.174 (-0.35 to -0.01) | -0.310 (-0.48 to -0.14) | -0.246 (-0.41 to -0.08) |
|  | **6 m Follow up** | -0.214 (-0.39 to -0.03) | -0.247 (-0.42 to -0.07) | -0.142 (-0.31 to 0.03) |
|  | **12 m Follow up** | -0.144 (-0.32 to 0.004) | -0.151 (-0.32 to 0.02) | -0.083 (-0.25 to 0.08) |
| **STIGMA** | | | | |
| **CAMI** | **Post-intervention** | -0.029 (-0.21 to 0.15) | 0.242 (0.07 to 0.42) | 0.495 (0.32 to 0.66) |
|  | **6 m Follow up** | 0.182 (0.00 to 0.36) | 0.342 (0.17 to 0.52) | 0.407 (0.24 to 0.58) |
|  | **12 m Follow up** | 0.064 (-0.12 to 0.24) | 0.166 (-0.01 to 0.34) | 0.275 (0.11 to 0.44) |
| **RIBS** | **Post-intervention** | -0.057 (-0.24 to 0.12) | 0.088 (-0.08 to 0.26) | 0.213 (0.04 to 0.38) |
|  | **6 m Follow up** | 0.017 (-0.16 to 0.20) | 0.092 (-0.08 to 0.26) | 0.155 (-0.01 to 0.32) |
|  | **12 m Follow up** | -0.033 (-0.21 to 0.15) | 0.017 (-0.16 to 0.19) | 0.174 (0.00 to 0.34) |
| **HELP SEEKING** | | | | |
| **Friend** | **6 m Follow up** | -0.138 (-0.32 to 0.04) | -0.045 (-0.22 to 0.13) | 0.054 (-0.11 to 0.22) |
|  | **12 m Follow up** | -0.122 (-0.30 to 0.06) | -0.043 (-0.22 to 0.13) | -0.026 (-0.19 to 0.14) |
| **Parent** | **6 m Follow up** | -0.122 (-0.30 to 0.06) | -0.077 (-0.25 to 0.10) | 0.037 (-0.13 to 0.21) |
|  | **12 m Follow up** | -0.104 (-0.28 to 0.08) | -0.080 (-0.25 to 0.09) | 0.019 (-0.15 to 0.19) |
| **Teacher** | **6 m Follow up** | -0.071 (-0.25 to 0.11) | -0.016 (-0.19 to 0.16) | 0.115 (-0.05 to 0.28) |
|  | **12 m Follow up** | -0.063 (-0.24 to 0.12) | -0.055 (-0.23 to 0.12) | 0.057 (-0.11 to 0.22) |
| **Mental health professional** | **6 m Follow up** | -0.229 (-0.41 to -0.05) | -0.116 (-0.29 to 0.06) | -0.085 (-0.25 to 0.08) |
|  | **12 m Follow up** | -0.203 (-0.38 to -0.02) | -0.074 (-0.25 to 0.10) | -0.047 (-0.21 to 0.12) |
| **No one** | **6 m Follow up** | -0.144 (-0.32 to 0.04) | -0.013 (-0.19 to 0.16) | 0.021 (-0.15 to 0.19) |
|  | **12 m Follow up** | -0.195 (-0.37 to -0.01) | 0.055 (-0.12 to 0.22) | 0.034 (-0.13 to 0.20) |

***Abbreviations:*** **CAMI** Scaling Community Attitudes toward the Mentally Ill; **EMHL** EspaiJove Mental Health Literacy Test; **MHL** Mental Health Literacy Programme; **MHL+SR** Mental Health Literacy Programme plus Stigma Reduction; **RIBS** Reported and Intended Behaviour Scale; **SP** Sensitivity Programme; **CI** Confidence Interval

***Cohen’s** : Interpretation effect sizes: small (0.2-0.5), moderate (0.5-0.8) and large (> 0.8).
